# The effect of maternal food insecurity transitions on housing insecurity in a population-based sample of mothers of young children

**DOI:** 10.3934/publichealth.2022001

**Published:** 2021-11-04

**Authors:** Erin Nolen, Catherine Cubbin, Mackenzie Brewer

**Affiliations:** 1 Steve Hicks School of Social Work, University of Texas at Austin, Austin, TX, USA; 2 Dell Medical School, Department of Population Health, University of Texas at Austin, Austin, TX, USA; 3 Department of Sociology, Baylor University, Waco, TX, USA

**Keywords:** food insecurity, housing insecurity, social cohesion, social support, population-based

## Abstract

**Background:**

Studies have shown a link between food insecurity and housing problems, including trouble paying rent. Additional research is needed to test the longitudinal effect of food insecurity on housing insecurity in a socio-demographically diverse, population-based sample. We tested whether food insecurity transitions predicted housing insecurity using a housing insecurity index consisting of housing and neighborhood factors. We also tested whether social cohesion or social support mediated the food/housing insecurity relationship.

**Method:**

Data were analyzed from a sample of 2868 mothers of young children residing in California at two time points: the baseline Maternal and Infant Health Assessment (2003–2007) and follow-up Geographic Research on Wellbeing survey (2012–2013). Women were categorized as food insecure both times; became food insecure; became food secure; and food secure both times. We constructed linear regression models for housing insecurity: models regressing each variable separately; a model regressing sociodemographic covariates and food insecurity status; mediation models adding social cohesion or social support; and mediation models for each racial/ethnic group.

**Results:**

Food insecurity transitions were associated with housing insecurity in a gradient pattern. Compared to women who were food secure both times, housing insecurity was highest among women who were food insecure both times, followed by those who became food insecure, and then those who transitioned out of food insecurity (became food secure). Food insecurity remained a significant risk factor for housing insecurity even after adjusting sociodemographic covariates. While social support and social cohesion were negatively associated with housing insecurity, there was limited evidence that social support/cohesion mediated the food insecurity/housing insecurity relationships.

**Conclusions:**

The lack of substantial mediation suggests that factors beyond social ties may explain the food and housing insecurity relationship. Efforts to reduce material hardship should consist of streamlined policy efforts that offer tangible supports for women and their families.

## 1. Introduction

### 1.1. Food insecurity

Food insecurity is a socioeconomic condition characterized as uncertain or limited access to adequate food [1]. While often conflated with the physiological condition of hunger, food insecurity represents a household's limited food resources and options typically due to financial constraint or reduced access to food [1]. The U.S. Department of Agriculture measures the prevalence of household-level food insecurity in the U.S. annually. In 2019, 10.5 percent of U.S. households were food insecure at varying points during the year. Single-mother households with children exhibit the highest rate of food insecurity (28.7%) across all demographic groups, and Black/non-Hispanic households (19.1%), and Hispanic households (15.6%) are more likely to report food insecurity compared to White households (7.9%) [1].

Parents in food-insecure households navigate a series of expense trade-offs to put food on the table [2]. These trade-offs are based on constrained, albeit rational, choices that are problem-solving methods or coping mechanisms for managing a limited food supply. For example, a mother may forgo a meal so a child can eat, share resources with family and friends, go to food banks/pantries, pawn personal possessions, skip housing or electric bills, or sacrifice housing and/or neighborhood quality to ensure the family is fed [2],[3]. Caregivers who make these trade-offs are at increased risk of “toxic stress” [3] and poor mental health [4].

Food insecurity has traditionally been measured at the household-level, so understanding the maternal-level impacts of food insecurity has been limited. Given that caregivers may bear the burden of skipping meals to ensure their children or other household members do not go without [2], food insecurity may be differentially experienced by family members within a household. Further, some evidence suggests that globally, women are more likely than men to experience food insecurity, underscoring a food-insecurity “gender gap” that is explained by “lower educational attainment, lower household income, and fewer social networks among women relative to men” [5]. For these reasons, women, particularly single mothers and mothers of color, may be at increased risk of food insecurity and other material hardships compared to men.

### 1.2. Food insecurity and housing issues

Given the trade-off realities and material hardship of households with food insecurity, studies have demonstrated a relationship between food insecurity and a variety of housing problems, including trouble paying rent. For example, inability to pay electric bills [6], housing costs greater than 30 percent of household income, being behind on rent, living in low quality living conditions [7], crowding, and multiple moves are housing factors associated with food insecurity [8]. Further, Sandel and colleagues [12] found that housing hardships (as measured by being behind on rent, multiple moves, or current homelessness) were associated with higher odds of food insecurity in low-income families who rent housing. While food insecurity is measured with a validated scale, housing insecurity lacks a gold standard measure. As a result, studies employ a range of housing-related indicators (intended to measure “housing insecurity” or “housing instability”), all of which are important in their own right, but lack sufficient coverage of the housing insecurity construct, such as focusing on affordability or missed payments at the expense of also measuring factors that impact housing livability, such as neighborhood quality and safety [9].

Prior work also found a reverse association between food and housing insecurity. Using data from the Fragile Families and Child Well-being study (which oversamples single mothers), King [10] found that food insecurity predicted housing instability, utilizing a more robust set of housing instability indicators including missed rent or mortgage payment, doubling-up, frequent moving, eviction, or homelessness. Additionally, Huang and King [11], using longitudinal data from Fragile Families (two time points, two years apart), demonstrated that families who were food insecure at both time points were at increased risk of housing instability compared to families that transitioned out of food insecurity from time 1 to time 2, underscoring the need to assess food security stability or transition in relation to housing instability. Indeed, the authors suggest that “food insecurity may be an early sign of economic hardship” [11]. While food insecurity may be short-term or cyclical (experienced in tandem with income volatility or economic recessions), families that experience persistent food insecurity are at increased risk of housing problems [11]. The authors also [11] found that food-insecure immigrant mothers are at increased risk of housing instability compared to non-immigrant mothers.

These studies lay an important foundation for food and housing insecurity research, including the importance of examining the effect of food insecurity transitions on material hardship. Testing these associations in a population-based sample is needed, particularly because families with income above the federal poverty level can still experience food insecurity, and some families below the poverty level are able to stave off food insecurity [1].

### 1.3. Social cohesion

In addition to being a household resource problem, food insecurity is a sociocultural phenomenon which is influenced by the social and physical environment [13]. Social cohesion represents the social connections in a neighborhood which promote trust, solidarity, and the ability to achieve common goals [14]. Denney and colleagues [13] built on existing evidence on the relationship between social cohesion and food insecurity [15]–[20] and found that social cohesion is protective against food insecurity in a population-based sample of mothers of young children (Geographic Research on Well-being Study), particularly for Black and Hispanic households. Denney and colleagues point to the role of informal networks, social support, and neighborly trust in helping families mitigate material constraints [13]. However, after hypothesizing that a lack of social cohesion might partially explain the relationship between food and housing insecurity, Huang and King [11] did not find that social cohesion significantly reduced the size of the relationship between food insecurity and housing instability in a sample of mothers who participated in the Fragile Families and Child Well-being Study. Therefore, while social cohesion may protect against the onset of food insecurity, additional evidence on whether social cohesion (or lack thereof) explains the food/housing relationship among a population-based, racially/ethnically diverse sample is warranted.

### 1.4. Social support

Further, food-insecure families that lack social support (such as the ability to borrow money from family and friends or to reach out to someone if they have an emergency), may be at increased risk of housing problems. There is some evidence that social support marginally explains the food/housing relationship: King [10] found that “food insecurity increases the risk of housing instability through a combination of material hardship and a lack of instrumental support”, though material hardship was the primary mediator (p. 258). Huang and King [11] found that while social support reduced the size of the relationship between food insecurity and housing instability, it was not significant. The authors suggest that, similar to social cohesion, families that are already food insecure may lack necessary social supports which may put them at greater risk of other aspects of material hardship, like housing insecurity [10],[11]. Again, additional evidence needs to test this association in a socioeconomically representative sample.

### 1.5. Present study

Given that food insecurity may be an early indicator of material hardship, we tested the conceptual model of Huang and King [11], who tested the effect of food security transitions over time on housing insecurity and whether social cohesion or social support explain the relationship. This model was particularly relevant to test with longitudinal data which spans the U.S. Great Recession because fluctuations in food insecurity are particularly sensitive to income volatility [21]. To build on the existing evidence above [10],[11], we assessed the mother's food insecurity transition (as opposed to the household's), used a housing insecurity index that consists of both housing problems and neighborhood safety per recent recommendations on housing insecurity measurement [9], and used a population-based sample of mothers of young children in the state of California (which is representative based on key sociodemographic variables including income, race/ethnicity, and marital status) to enhance generalizability. In addition to the primary aims listed above, we conducted a sensitivity analysis to determine if there were differences in the food/housing insecurity associations by racial/ethnic group. Food insecurity is especially high among Latino immigrant families, [22] so we stratified Latinas by nativity.

## 2. Materials and methods

### 2.1. Data source

This study is based on the 2003–2007 Maternal and Infant Health Assessment (MIHA) and its follow-up survey, the 2012–2013 Geographic Research on Wellbeing (GROW) study. MIHA is California's version of CDC's Pregnancy Risk Assessment Monitoring System (https://www.cdc.gov/prams/index.htm) and is an annual, statewide-representative survey of mothers delivering live infants in California during February through May, linked with birth certificate data, which serves as its sampling frame [23],[24]. Out of approximately 500,000 births per year, MIHA collected data from a representative sample of 3500 mothers using a questionnaire that was administered by mail or phone, in English (71%) or Spanish (29%); response rates exceeded 70% each year. The maternal characteristics of the MIHA sample are weighted to be representative of all eligible births statewide.

MIHA respondents had the option to agree to be re-contacted for future studies; 95% of them said yes. Among those, if they lived in one of six large, urbanized counties at the time of the 2003–2007 surveys, they were eligible for GROW (representing 55% of all MIHA respondents from 2003–2007). Data collection for GROW took place from 2012–2013. The questionnaire comprised approximately 80 questions regarding demographic, socioeconomic, neighborhood, psychosocial, and health-related characteristics. They received a gift card and a chance to win raffles as incentives.

Of the 9256 women who were initially identified as eligible to be in the GROW sample, 3016 responded (32.6%); of the 4026 women who could be located (“active” sample), 74.9% responded. The large majority of respondents (90.3%) still lived in one of the six GROW counties. Fifty-six percent completed the survey by phone (vs. 44% by mail), and 73% completed it in English (vs. 27% in Spanish). Missing values for income (9.8%) were imputed using hot-deck methodology and multiple sociodemographic variables. Weights were created to produce data that were representative of births in the six GROW counties, and a sampling fraction file was created to make a minor finite population correction to the standard errors for analyses [25]. The analytic dataset included women whose race/ethnicity was self-reported as African American, Asian or Pacific Islander, Latina, or White. If any cases had missing data on the variables of interest, they were excluded from the analysis, resulting in 2837 records (94%). The GROW study was approved by the Institutional Review Boards at the University of Texas at Austin, the University of California, San Francisco, and the California Department of Public Health; all participants gave informed consent. Additional details about the GROW study have been reported [25].

### 2.2. Variables

The dependent variable was housing insecurity at time of follow-up in the GROW (2012–2013) study, measured as a sum of 9 questions on housing insecurity-related questions, including stressors during the past year (had to move because of problems paying the rent or mortgage, did not have a regular place to sleep at night, being homeless), renter, moved more than one time in the past five years, moved to her current neighborhood because it was all she could afford, and neighborhood safety (had anything stolen from inside her home, been a victim of or seen violence, neighborhood is somewhat or very unsafe). We choose the term “housing insecurity” over “housing instability” (though often used interchangeably) to remain consistent with the recommendations of Cox and colleagues [9].

The independent variable was food insecurity, which represented the mother's food insecurity status at two time points: during pregnancy at MIHA (baseline) and during the previous 12 months at GROW (follow-up). Food insecurity was measured from the 6-item Household Food Security scale developed and validated by researchers at the National Center for Health Statistics [26]. Although the 6-item short form is a household-level measure of food insecurity, it was modified for the purpose of the MIHA and GROW survey program which was to estimate the mother's food security status during pregnancy and at follow-up. Items were modified by referencing the mother's direct experience of food insecurity instead of the household altogether to develop an individual-level proxy estimate of food insecurity. Examples of items include, “The food I bought just didn't last, and I didn't have money to get more”, “I couldn't afford to eat balanced meals”, and “Did you ever cut the size of meals or skipped meals because there wasn't enough money for food?” We coded mothers who answered affirmatively to at least two of the items as food insecure [26] at either time point and then created a variable with four categories to determine food insecurity transitions (food insecure both times; became food insecure; became food secure; food secure both times).

The two other variables of interest were perceived neighborhood social cohesion and perceived individual social support, that we conceptualized as potential mediators between food insecurity status and housing insecurity. Social cohesion was measured at GROW (follow-up) following the method of Sampson et al. [14] using five questions asking agreement with the following statements: “My neighbors feel connected to each other”, “People in my neighborhood are willing to help their neighbors”, “People in my neighborhood generally get along with each other”, “People in my neighborhood share the same values” and “People in my neighborhood can be trusted”. Modifications to Sampson et al. [14] included slight variations in wording. For example, in GROW, “my neighbors feel connected to each other” was used instead of the original wording, “this is a close-knit neighborhood” because of concerns that some immigrants would not understand “close-knit”. In addition, instead of “in this neighborhood”, GROW used “in my neighborhood”. Responses range from strongly agree (4) to strongly disagree (1), while Sampson et al. [14] used a 5-point Likert scale. The original items have been demonstrated to have high internal consistency and test-retest reliability among a sample of women [27]. For GROW respondents with at least 3 of the 5 items answered (86% of the sample), we averaged the values, and standardized the measure. The mean value (14.72, higher values indicate more cohesion) was imputed for respondents with missing data (i.e., those who did not respond to at least three of the five questions.

Social support was measured at GROW (follow-up) with three yes/no questions: “Do you have someone you could turn to if you needed ... someone to comfort or listen to you?; ...practical help, like getting a ride somewhere, help with shopping or cooking a meal, or help watching your children for a short time?; ... some extra help financially, like help paying for some bills, the rent or mortgage, or food that you needed?” High social support indicated an affirmative answer to all three.

Other variables included age (20–29 years, 30–39 years, 40 years and older), race/ethnicity and nativity status (non-Hispanic African American, non-Hispanic Asian or Pacific Islander, Latina U.S.-born, Latina immigrant, non-Hispanic White), marital status (married or cohabitating, not married or cohabitating), number of children in the household (0–1, 2–3, 4 or more), educational attainment (did not complete high school, high school graduate/GED, some college, college graduate), and income (annual family income, in increments of the federal poverty level: 0–100%, 101–200%, 201–400%, 401%+). Nativity status was pulled from birth certificate data. We conducted analyses with Latina women split by nativity status only because other race/ethnicity groups did not have sufficient sample sizes when split by nativity status.

### 2.3. Analyses

**Figure 1.**
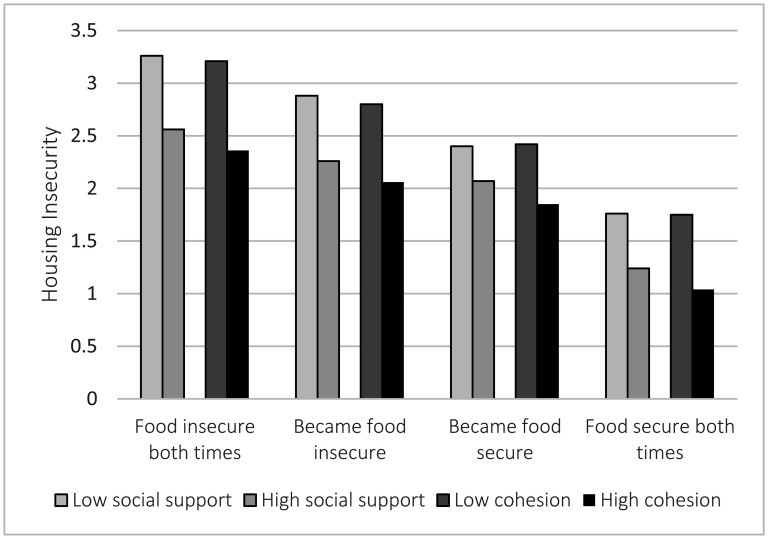
Housing insecurity index by food security status and social support/cohesion, geographic research on well-being survey, *N* = 2,837. Note: Social cohesion was split at the median value (15).

For descriptive analyses, we first examined the distribution of all variables (Table 1). Next, we examined bivariate relationships between the independent (food insecurity transitions) and dependent (housing insecurity) variable stratified by our potential mediators, social support and cohesion (Figure 1). Finally, we constructed a series of linear regression models for housing insecurity: (1) “crude” models regressing each variable separately; (2) a “sociodemographic” model regressing age, race/ethnicity and nativity status, marital status, number of children in the household, educational attainment, income, and food insecurity transitions on housing insecurity; (3) two “mediation” models adding social support or social cohesion to the sociodemographic model (Table 2). Preliminary analyses determined that both mediator candidates were associated with food insecurity transitions and housing insecurity. If the coefficients in the mediation models are substantially reduced or eliminated when compared to the sociodemographic model, we will conclude as evidence of mediation [28]. Finally, as a sensitivity test, we constructed the mediation models for each racial/ethnic group to examine whether the associations of interest differed by race/ethnicity (including nativity status for Latinas). Social cohesion was centered at the mean. All analyses were conducted using SAS version 9.4 (Cary, NC) and incorporated weighting and adjusted for the complex sample design.

## 3. Results

Nearly half of the women were between ages 30–39 and over half were Latina (Table 1). The large majority were married or cohabitating and about two-thirds of households had 2–3 children living in them. One-fifth had not completed high school while over one-third were college graduates. Over half of the women had incomes that were at or below two times the federal poverty level, while about 30% of women had incomes that were over four times the federal poverty level. Women reported high social support (75%), relatively high social cohesion (mean 14.7 in a range from 5–20), and relatively low housing insecurity (1.7 in a range of 0–9). At baseline, food insecurity was 17% (MIHA, 2003–2007; pre-Great Recession) climbing to 23% 5–10 years later at follow-up (GROW, 2012–2013; post-Great Recession). There was some change in food insecurity over the time frame with 14% moving from food secure to food insecure and 8% moving from food insecure to food secure.

For each social support/cohesion group, women had the highest housing insecurity if they were food insecure both times, and the lowest housing insecurity if they were food secure both times (Figure 1). Those who became food insecure had higher levels of housing insecurity than those who became food secure over the time frame. At each level of food insecurity, housing insecurity was higher for women with low vs. high social support and for women with low vs. high social cohesion (based on median split).

**Table 1.** Descriptive characteristics, geographic research on well-being survey, *N* = 2837.

	% or mean (range)
Age, in years	
20–29	18.5
30–39	49.2
40+	32.2
Race/ethnicity	
African American	6.6
Asian or Pacific Islander	15.1
Latina, US-born	16.1
Latina, immigrant	36.5
White	25.8
Marital status	
Married/co-habitating	83.4
Not married/co-habitating	16.6
Number of children in household	
0–1	11.2
2–3	64.5
4+	24.3
Educational attainment	
Did not complete high school	19.9
High school graduate/GED	22.7
Some college	22.8
College graduate	34.5
Income, as % of federal poverty level	
≤100%	31.5
101–200%	20.5
201–400%	18.9
>400%	29.1
High Social support (yes)	74.6
Social cohesion	14.73 (5–20)
Food insecure, baseline (2003–2007)	17.2
Food insecure, follow-up (2012–2013)	23.1
Food insecure both times	9.6
Became food insecure	13.8
Became food secure	7.6
Food secure both times	69.4
Housing insecurity	1.65 (0–9)

Note: GED = General Education Development test.

Table 2 presents the results of the linear regression models. In the crude models, each variable was associated with housing insecurity. Being younger, African American or Latina, not married/not cohabitating, in a household with 4 or more children, of lower education, and with lower income had higher risk of housing insecurity compared with women who were older, White, married/cohabitating, in a household with fewer children, college graduates, and with incomes over four times the poverty level. Asian or Pacific Islander women were not significantly different in housing insecurity compared with White women. Higher social support and social cohesion were associated with lower risk of housing insecurity, and all groups of food insecurity transitions had higher risk of housing insecurity in a gradient pattern compared with those who were food secure both times.

Food insecurity transitions remained statistically significant in the sociodemographic model adjusted for age, race/ethnicity and nativity status, marital status, number of children in the household, educational attainment, and income. Moreover, aside from U.S.-born Latinas (which became insignificant), all of the other variables remained significant but attenuated. Although the food insecurity associations with housing insecurity were attenuated with either social support or cohesion in the model, they remained substantial and significant, suggesting little evidence of mediation. However, African American women's increased risk become statistically similar to White women in both mediation models.

In supplemental analyses of the mediation models stratified by race/ethnicity, although not all findings were significant at conventional levels due to smaller sample sizes, the direction of the relationships between all the variables and housing insecurity were similar (results available upon request).

**Table 2.** Linear regression of housing insecurity, geographic research on well-being survey, *N* = 2,837.

	Crude	Sociodemographic	Mediation/High social support	Mediation/Social cohesion
Age, in years				
20–29	1.00 (0.08) ***	0.30 (0.08) ***	0.32 (0.08) ***	0.24 (0.08) ***
30–39	0.50 (0.06) ***	0.17 (0.05) ***	0.18 (0.05) ***	0.16 (0.05) ***
40+	Reference	Reference	Reference	Reference
Race/ethnicity				
African American	1.02 (0.10) ***	0.19 (0.10) *	0.18 (0.10)	0.13 (0.10)
Asian or Pacific Islander	−0.04 (0.07)	0.02 (0.08)	0.02 (0.07)	−0.04 (0.07)
Latina, US-born	0.59 (0.09) ***	−0.01 (0.08)	−0.01 (0.08)	−0.05 (0.08)
Latina, immigrant	1.30 (0.07) ***	0.31 (0.00) ***	0.29 (0.09) **	0.24 (0.09) **
White	Reference	Reference	Reference	Reference
Marital status				
Married/co-habitating	Reference	Reference	Reference	Reference
Not married/co-habitating	0.89 (0.09) ***	0.35 (0.08) ***	0.34 (0.08) ***	0.33 (0.08) ***
Number of children in household				
0–1	Reference	Reference	Reference	Reference
2–3	−0.13 (0.09)	−0.13 (0.08)	−0.12 (0.08)	−0.09 (0.08)
4+	0.31 (0.11) **	−0.20 (0.09) *	−0.17 (0.09)	−0.17 (0.09)
Educational attainment				
Did not complete high school	1.50 (0.07) ***	0.30 (0.10) **	0.25 (0.10) *	0.26 (0.10) **
High school graduate/GED	1.30 (0.07) ***	0.23 (0.09) *	0.22 (0.09) *	0.20 (0.09) *
Some college	0.82 (0.07) ***	0.21 (0.07) **	0.21 (0.07) **	0.14 (0.06) *
College graduate	Reference	Reference	Reference	Reference
Income, as % of FPL				
≤100%	1.76 (0.06) ***	0.81 (0.10) ***	0.80 (0.10) ***	0.70 (0.10) ***
101–200%	1.39 (0.07) ***	0.69 (0.09) ***	0.67 (0.09) ***	0.60 (0.09) ***
201–400%	0.60 (0.06) ***	0.26 (0.06) ***	0.26 (0.06) ***	0.20 (0.06) **
>400%	Reference	Reference	Reference	Reference
High social support	−0.95 (0.08) ***		−0.37 (0.07) ***	
Social cohesion	−0.22 (0.01) ***			−0.13 (0.01) ***
Food insecurity transitions				
Food insecure both times	1.74 (0.12) ***	1.05 (0.12) ***	0.92 (0.12) ***	0.91 (0.11) ***
Became food insecure	1.29 (0.09) ***	0.74 (0.09) ***	0.66 (0.09) ***	0.64 (0.09) ***
Became food secure	0.93 (0.09) ***	0.33 (0.09) ***	0.30 (0.09) **	0.30 (0.08) ***
Food secure both times	Reference	Reference	Reference	Reference

Note: GED = General Education Development test; FPL = Federal Poverty Level; *p < 0.05; **p < 0.01; ***p < 0.001.

## 4. Discussion

This study contributes to the food and housing insecurity literature by utilizing a population-based sample of mothers of young children in California and employing a housing insecurity index that incorporates housing and neighborhood indicators. Food insecurity and lack of decent, affordable housing are persistent problems in the United States. Rates of food insecurity peaked at 14.6 percent in 2011 following the Great Recession and remained higher than pre-recession (2007) levels until 2018. The onset of the COVID-19 pandemic and economic recession upended recent declines, with an estimated 45 million people (1 in 7) experiencing food insecurity in 2020 [29]. Individuals experiencing food insecurity often struggle to meet other basic needs including stable, adequate housing. The data in this study consisted of two time points which spanned the Great Recession (2003–2007 at time 1 and 2012–2013 at time 2). The longitudinal patterns in material hardship over this time period may have implications for the repercussions of economic decline caused by the COVID-19 pandemic.

With a robust housing insecurity measure and using a population-based dataset, this study's findings corroborate previous research and demonstrate a link between food insecurity and housing problems [6]–[8],[10],[11],[19],[30],[31]. The findings indicate a gradient pattern where persistently food insecure mothers had the highest risk of housing insecurity followed by mothers who became food insecure. Thus, even transient states of food insecurity were associated with housing instability independent of other risk factors, such as low-income and lack of education. Households with limited financial resources often make difficult trade-offs in expenses to secure basic needs [32]. Our findings are in line with Huang and King [11] who found that families who experience enduring food insecurity over time are more likely to experience housing-related problems, including difficulty in paying rent or utility bills.

This study also assessed whether social cohesion and social support mediated the food insecurity/housing insecurity relationship. Previous studies found that social cohesion is protective against food insecurity [13],[15]–[17],[20],[33], but none of these studies incorporated housing insecurity as the dependent variable. Unlike the current study, Huang and King [11] found no association between social cohesion and housing insecurity after accounting for a host of individual and neighborhood-level risk factors, though their sample was limited in generalizability. The current study's findings showed that low social cohesion was associated with high housing insecurity but did not substantially mediate the relationship between food and housing insecurity among a sample of women with children who spanned the socioeconomic spectrum.

The findings were similar for social support: high support was associated with lower housing insecurity, but did not serve as a substantial mediator of the food/housing insecurity relationship. Among a sample of lower-income, mostly unmarried mothers, King [10] found that a lack of instrumental support marginally mediated the link between food and housing insecurity, suggesting that reduced social connections among food insecure families may contribute to housing problems (and, in turn, higher social support may help reduce such problems). Nonetheless, material hardship, indicated through various dimensions of economic constraint (e.g., an inability to pay electric bills or seek medical care because of cost), accounted for almost half of the association between food and housing insecurity, demonstrating the importance of policies and public assistance programs that address food and housing needs [10]. Additionally, because housing is such a large expense, it is possible that even among those families who do have social support, those connections may not be sufficient for supporting their financial constraints.

Taken together, the present study's findings offer limited evidence that social networks, operating at the household and neighborhood-level, can explain the relationship between food and housing insecurity. This suggests that while food insecure mothers may lack strong social networks, other factors beyond social ties may better explain how food insecurity predicts housing insecurity among mothers. Moreover, in-depth interviews with low-income parents illustrate how the receipt of instrumental support from social ties (e.g., moving in with family to alleviate housing instability) can actually be a source of stress with significant emotional costs for recipients [34]. Thus, it is important to address the social and financial needs of households experiencing forms of material hardship, such as food and housing insecurity.

Our study makes several contributions to the literature. Previous studies investigating food and housing insecurity utilized primarily low-income and/or non-population-based samples and thus are limited in terms of socioeconomic representativeness. While households in poverty are more likely to experience food insecurity, many families above the poverty threshold still experience food insecurity [35]. Conversely, not all families below the poverty threshold are food insecure [1],[35]. Our sample is representative of mothers of young children residing in California based on other key socio-demographic variables, including race/ethnicity and marital status. Given this reality, examining data from a socioeconomically representative sample allows for greater external validity. Moreover, this study estimated maternal food insecurity to quantify women's specific experiences of food insecurity and its relationship with other individual-level factors, including perceived social cohesion and social support.

Further, this study's index of housing insecurity included indicators related to housing stability, housing affordability, neighborhood safety, and homelessness, which are four of seven domains recommended for ensuring a more comprehensive assessment of housing insecurity [9]. While we were not able to capture the other dimensions (neighborhood quality, housing quality and housing safety), few studies incorporate neighborhood-related factors in housing insecurity assessments. Cox, et al. [9] found that only 8 percent of papers addressing housing insecurity used at least four of the seven recommended domains and have since pressed for a uniform definition and a national standardized measure, not unlike that of the U.S. Department of Agriculture's Food Security Module, to better understand the prevalence and impact of housing insecurity in the U.S. Comprehensive measures ensure families who have a variety of housing or neighborhood problems are considered in data, policy, and program solutions.

Strengths notwithstanding, several limitations are of note. The 6-Item Household Food Insecurity scale was modified by the MIHA and GROW survey administrators to estimate the mother's individual-level experience of food insecurity. We recognize it is difficult to parse out individual-level experiences of food insecurity from the household-level experience given that households share resources. Future research might consider utilizing the Food Insecurity Experience Scale designed to measure individual-level food insecurity [36]. Furthermore, while this study examined the effect of food insecurity transitions on housing insecurity, Lee et al. [31] provide evidence for the bi-directional relationship between food insecurity and housing instability using cross-lagged path analyses of two time points of data from the Fragile Families and Child Well-being Study. The authors [31] suggest that financial constraint puts families at risk of experiencing food insecurity and housing instability, which are correlated, yet distinct forms of economic hardship. It is possible that housing problems (particularly over time) may exacerbate food insecurity (e.g., mothers may cut back on food expenses at the end of the month if she is having difficulty getting her rent paid on time and wants to avoid future eviction). We were not able to test transitions in housing situations with the current data because the housing insecurity items at follow-up (GROW) were not asked at baseline (MIHA). While these findings are representative of mothers of young children in the state of California, they cannot be generalized to the national population. Future research should continue to test these relationships longitudinally and within larger, population-based samples to gain sufficient power for analyses.

The survey is also based on self-reported information which may introduce bias. For example, women with housing insecurity may be more likely to endorse lower levels of perceived neighborhood social cohesion. Because the survey was conducted in English and Spanish, non-English-speaking Asian participants were under-represented. While food insecurity disproportionately impacts women, it particularly affects women and households of color. In sensitivity tests we found no evidence that the food/housing insecurity association varied across racial and ethnic groups and nativity status (stratifying Latinas by nativity). Future research should continue exploring whether risk and protective factors related to housing insecurity are moderated by intersectional factors such as race, ethnicity, social class, and sexual orientation.

Further, we lack information on other factors that might shape the food/housing insecurity relationship, such as intimate partner violence [37],[38]. Additionally, while our housing insecurity index was encompassing of housing indicators to capture a variety of housing problems that may be overlooked in current research, this study did not tease out differences by indicator, so whether homelessness or other forms of housing insecurity largely account for the effect of food insecurity transitions on housing insecurity was not tested.

## 5. Conclusions

Housing and food are fundamental human rights which affect the health and well-being of women and their families. Tangible supports for women that might reduce constrained decision-making and temporarily ameliorate stretched household budgets include food assistance programs such as Supplemental Nutrition Assistance Program (SNAP) and the Special Supplemental Nutrition Program for Women, Infants, and Children (WIC) program. Further, it is crucial that screening and providing referrals for housing and food assistance go hand in hand in social service and health care settings [39]. However, while vetted screening tools exist for food insecurity [40], tools for housing insecurity are limited, and service providers and health professionals should screen for factors beyond homelessness and include other housing and neighborhood factors, including safety and quality. [9]. Finally, efforts to address food insecurity at the policy level could also consider housing policy solutions, such as universal voucher programs and organizing free legal support for unjust foreclosures, evictions, or compromised housing quality and safety [41].
